# Polyaniline/ZnO Hybrid Nanocomposite: Morphology, Spectroscopy and Optimization of ZnO Concentration for Photovoltaic Applications

**DOI:** 10.3390/polym15020363

**Published:** 2023-01-10

**Authors:** Alamgeer, Muhammad Tahir, Mahidur R. Sarker, Shabina Ali, Ibraheem, Shahid Hussian, Sajad Ali, Muhammad Imran Khan, Dil Nawaz Khan, Rashid Ali, Suhana Mohd Said

**Affiliations:** 1 Department of Physics, Faculty of Physical and Numerical Sciences, Abdul Wali Khan University Mardan, Mardan 23200, Pakistan; 2 Institute of IR 4.0, Unverisity Kebangsaan Malaysia, Bangi 43600, Malaysia; 3 Faculty of Materials and Chemical Engineering, Ghulam Ishaq Khan Institute of Engineering Sciences and Technology, Topi 23460, Pakistan; 4 Pak-Austria Fachhochschule: Institute of Applied Sciences and Technology, Haripur 22620, Pakistan; 5 Department of Electrical Engineering, Faculty of Engineering, University of Malaya, Kuala Lumpur 50603, Malaysia

**Keywords:** polyaniline, Zinc Oxide, nanocomposite thin film, I-V characteristics, heterojunction solar cell, optical band gap

## Abstract

The appropriate combination of semiconducting polymer–inorganic nanocomposites can enhance the existing performance of polymers-only-based photovoltaic devices. Hence, polyaniline (PANI)/zinc oxide (ZnO) nanocomposites were prepared by combining ZnO nanoparticles with PANI in four distinct ratios to optimize their photovoltaic performance. Using a simple coating method, PANI, ZnO, and its nanocomposite, with varying weight percent (wt%) concentrations of ZnO nanoparticles, i.e., (1 wt%, 2 wt%, 3 wt%, and 4 wt%), were fabricated and utilized as an active layer to evaluate the potential for the high-power conversion efficiency of various concentrations, respectively. PANI/ZnO nanocomposites are characterized by X-ray diffraction (XRD), scanning electron microscope (SEM), atomic force microscopy (AFM), Fourier transform infrared (FTIR) spectroscopy, ultraviolet-visible (UV-vis) absorption, energy dispersive X-ray (EDX), and I-V measurement techniques. The XRD analysis showed a distinct, narrow peak, which corresponds to the wurtzite ZnO (101) plane. The SEM analysis verified the production of the PANI/ZnO composite by demonstrating that the crystalline ZnO was integrated into the PANI matrix. The elemental composition was determined by energy dispersive X-ray analysis (EDX), which confirmed the existence of PANI and ZnO without any impurities, respectively. Using Fourier transform infrared (FTIR) spectroscopy, various chemical bonds and stretching vibrations were analyzed and assigned to different peaks. The bandgap narrowing with an increasing PANI/ZnO composition led to exceptional optical improvement. The I-V characterization was utilized to investigate the impact of the nanocomposite on the electrical properties of the PANI/ZnO, and various concentrations of ZnO (1 wt%, 2 wt%, 3 wt%, and 4 wt%) in the PANI matrix were analyzed under both light and dark conditions at an STC of 1.5 AM globally. A high PCE of 4.48% was achieved for the PANI/ZnO (3 wt%), which revealed that the conductivity of the PANI/ZnO nanocomposite thin films improved with the increasing nanocomposite concentration.

## 1. Introduction

Since the concern over the world’s energy demand and consumption grows, researchers are focusing on exploring alternate and renewable energy sources. Renewable energy sources, including wind, hydro, and solar, have been examined as potential substitutes for restricted and pollution-creating fossil fuels [1,2]. However, hydroelectricity and wind energy are expensive technologies regarding their installation, limited geographical applications, and maintenance. On the other hand, sunlight shines almost equally everywhere on the earth’s crust, even in remote areas [3]. Hence, sunlight can be immediately utilized for power production by using photovoltaic (PV) devices and technology. During recent decades, silicon (Si)-based solar cells have prevailed in the PV market due to their stability, lifetime, and higher power conversion efficiencies (PCEs), which have reached up to 25% [4]. However, one of the key drawbacks of Si-based PV devices is the economical factor for consumers that these PV modules are highly expensive and, at the same time, from technological aspects, this technology requires high-temperature processing in inert environments, i.e., cleanrooms, which are too costly to maintain [5,6]. Also, Si-based PV modules are mechanically brittle and can be easily broken if they fall down due to wind or storm. Therefore, alternate materials—organic semiconductors—in place of inorganic materials are favored due to their versatility, low cost, and easy processing at low temperatures.

Amongst organic semiconductors, polymeric semiconductors are the best materials- due to their solubility in a variety of organic solvents that consequently lead to facile device fabrication methods, i.e., spin-coating, drop-casting, dip-coating, etc. [7,8]. Soluble polymeric semiconducting materials allow one to physically mix p-type and n-type polymers to form donor/acceptor (*D/A*) interfaces throughout the bulk of the blend. PV devices based on the polymers’ *D/A* configuration have several advantages over others, i.e., they absorb most of the solar spectrum and can be tuned to achieve the desirable properties of the devices easily. Although organic semiconductors have the aforementioned benefits, these materials still, however, have a couple of shortcomings in the form of slightly lower charge carriers’ mobility, PCE, and lifetime [9]. Researchers are trying to overcome these issues by using different hybrid materials and nanocomposites, as well as nanostructures and device architectures. For this reason, recently, organic–inorganic nanocomposite systems have received significant attention for the enhanced performance of electronic and optoelectronic devices [10]. These types of hybrid nanocomposites possess both the characteristics of polymeric materials (they play a role in the matrix material) and inorganic-nanostructured materials (as dopant materials) due to the higher surface-to-volume ratio of the inorganic nanomaterials. Recently, hybrid polymer–inorganic nanocomposite-based solar cells have attained a PCE of 12% [11]. Furthermore, organic solar cells made of conjugated polymers and metal oxide semiconductors, such as TiO_2_, ZnO, SnO_2_, etc., are capable of superfast photo-induced charge transfer [12,13], as compared to individual materials [14].

To address the issues with the only-polymers solar cells, a hybrid polymer–inorganic nanocomposite approach is proposed to enhance the PCE of polymer PV devices [15]. Amongst the variety of semiconducting polymers, polyaniline (PANI)—having p-type conductivity—exhibits interesting electronic and optoelectronic properties due to an extended π-conjugated electrons system. PANI demonstrates a relatively higher and broader absorption of the ultraviolet and visible spectrum of sunlight. Having good electrical properties, it is also comparatively highly stable at ambient conditions and less toxic than other organic materials [1]. Simultaneously, PANI has availability at low cost, mechanical flexibility, and facile processing for device fabrication due to its solubility in chloroform, p-xylene, m-cresol, etc. These are some of the remarkable characteristics that PANI has successfully found its potential applications in, in many electronic and optoelectronic devices, including solar cells [16], sensors, thermoelectric devices, etc. [14].

On the other hand, zinc oxide (ZnO) is an inorganic semiconductor crystal with n-type electrical conductivity and a wide optical bandgap (3.37 eV) [2]. ZnO is one of the most likable materials due to its easy synthesis, non-toxicity, significant optoelectronic characteristics, and high stability, as well as its low cost. However, the metal–organic framework (MOF) based on zinc has the ability to convert specifically into distinctive zinc oxide nanostructures inspired by the interconversion synthesis of zeolites.

Esmail Doustkhah et al. transformed MOF-5 into nanocrystalline ZnO and discovered that the process is easy, adjustable, and capable of being regulated by temperature treatment and the selection of the right structure-directing agent (SDA). Subsequently, researchers found that compared to other ZnO nanostructures made at lower temperatures, the 180 °C-produced ZnO exhibits higher photocatalytic activity in the degradation of methylene blue. They also discovered that the changed crystal development in ZnO leads to enhanced photocatalytic activity, while ZnO’s wide energy bandgap is not substantially affected by the hydrothermal approach used to alter the thermodynamically favored crystal orientation of ZnO nanocrystals [17]. Due to its high photocatalytic performance, ZnO has become the best candidate for a variety of potential uses, especially when it is in the form of nanowires, nanorods, nanoparticles, or as a thin film in solar cells [18,19,20]. Furthermore, ZnO is successfully utilized in organic solar cells as a buffer layer and/or electron transport layer (ETL) and also as a semi-transparent electrode. ZnO can form hybrid organic–inorganic bulk heterojunction (BHJ) solar cells wherein it acts as the electron-accepting material, while the organic semiconductor plays a role in the electron donor materials [21]. ZnO has been the subject of significant investigation because of its many technological uses, such as long-range ordering among magnetic ions, which should be mediated for optoelectronic devices by itinerant carriers with the proper density and mobility. Itinerant carriers in ZnO typically come from carrier co-doping or inadvertent point defects in the material. Particularly, the unintended impurities in ZnO play a crucial function in regulating the interactions in ZnO by drastically influencing the electrical activity of the material. For instance, ZnO has consistently displayed n-type conductivity, even when not intentionally doped. Initially, Van der Wall suggested that the H in ZnO would be positively charged during a large portion of the experimental bandgap, and as a result, this is a potential reason for the n-type behavior. Moreover, the incorporation of ZnO in a polymeric matrix can absorb inadvertent H-doping, resulting in a high carrier concentration, which is advantageous for solar applications. Several experiments later validated this prediction, as well [22]. However, similar to the significance of the choice of a suitable donor and acceptor materials, a particular concentration of the donor/acceptor materials is also important, which affects the interfacial area throughout the bulk available to the excitons for dissociation into separate electrons and holes. This can, consequently, tune and improve the excitons’ breaking and decrease the recombination processes that affect the PCE of solar cells [23]. Additionally, once the blending ratio of the donor/acceptor materials is properly tuned, then one can obtain optimum parameters of the processes that take place during the generation and separation of the excitons, as well as a collection of the charges at the corresponding electrodes.

In this work, we report the preparation of PANI/ZnO nanocomposite-based hybrid BHJ solar cells with different wt.% of ZnO nanoparticles in the PANI matrix. The different concentrations of ZnO in the PANI were employed in order to obtain the optimum ratio of the donor/acceptor combination for the improved PCE of the hybrid BHJ solar cells. A facile fabrication technique of spin-coating was used for the formation of ITO/P3HT/PANI/ZnO/Ag BHJ solar cells. The morphology and spectroscopy of the PANI/ZnO blend were studied by AFM, SEM, UV-Vis, and FTIR spectrophotometers. Moreover, the dark current–voltage (*I-V*) and PV characteristics of the fabricated ITO/P3HT/PANI/ZnO/Ag hybrid BHJ solar cells were measured at ambient conditions and standard test conditions (STC), respectively. Whereas the poly-3-hexylthiophene (P3HT) acted as a hole transport layer (HTL), owing to its outstanding hole mobility on the rigid and flexible substrate. From the *I-V* in the dark and PV measurements, it was observed that the ITO/P3HT/PANI/ZnO/Ag device performed better at 3 wt% of ZnO in the PANI/ZnO blend.

## 2. Materials and Methods

### 2.1. Device Preparation

**Figure 1:**
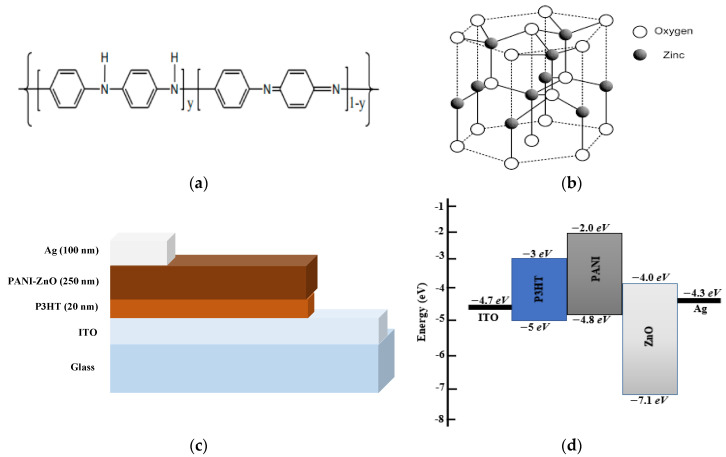
Molecular structure of (**a**) PANI and (**b**) ZnO (**c**) structure of fabricated ITO/P3HT/PANI/ZnO/Ag solar cell device and (**d**) band-bending diagram of the materials utilized in the device.

PANI, ZnO nanoparticles, and P3HT were received from Sigma-Aldrich and used as they were received. The average particle size of the ZnO nanoparticles was around 100 nm. The molecular structures of the PANI and ZnO are depicted in Figure 1a,b, respectively. The pre-patterned indium tin oxide (ITO) coated on the glass was used as a substrate for the bulk heterojunction solar cells, whereas quartz glass was used as the substrate for the PANI/ZnO nanocomposite thin films for its morphological and spectroscopic analysis. All the substrates were properly cleaned in acetone and isopropanol for 15 min each by using an ultrasonic bath (Elma E 300 H), followed by drying with nitrogen gas. On the cleaned ITO substrates, a thin layer (~20 nm) of P3HT was deposited from a chloroform solution using a spin coater (Fytronix Model No. FY-8000, Fytronix, Firat, Turkey), operating at 3000 rpm for a time of 20 s. Then, the samples were placed in a drying oven (FAITHFUL WHL-25AB, FAITHFUL Instrument Co., Ltd Cangzhou, China) for 2 h at 50 °C. Afterward, a uniform solution of PANI was formed in m-cresol at a concentration of 5 mg/mL, with the help of a magnetic stirrer. Furthermore, the ZnO nanoparticles were dispersed in m-cresol with different concentrations, i.e., 1 wt%, 2 wt%, 3 wt%, and 4 wt%, to form the optimized blend of PANI/ZnO nanocomposites. Moreover, the four different concentrated PANI/ZnO nanocomposite blends were separately spin-coated on the deposited P3HT/ITO samples at a speed of 3000 rpm for 20 s to fabricate four PANI/ZnO/P3HT/ITO devices with different wt.% of ZnO. The devices were kept in the drying oven to let the solvent evaporate from the films. Finally, a 100 nm layer of silver (Ag) was thermally deposited as the top electrode on the PANI/ZnO layer to form the ITO/P3HT/PANI/ZnO/Ag device. A vacuum thermal evaporator (model Osaka-YKY Inc.) at a vacuum level of 1.5 × 10^−5^ mbar was used for the thermal deposition of Ag with the in situ film thickness monitor at 2 nm/s. Figure 1c,d show the structure of the fabricated ITO/P3HT/PANI/ZnO/Ag device and the energy band diagram, respectively. The particle size (~100 nm in the present work) of ZnO is an important factor because the bandgap of the materials increases when the particle size decreases, which is the well-known quantum confinement effect. However, the HOMO and LUMO levels mentioned in Figure 1d express the bandgap information according to the given particle size.

### 2.2. Device and Thin Film Characterization

The optical bandgap and molecular bond dynamics of PANI/ZnO (1:1) by vol% were analyzed by using a Lambda 1050 UV-Vis spectrometer and PerkinElmer Frontier FTIR spectrometer, respectively. For morphological investigations of the thin films, a scanning electron microscopy (SEM) (Zeiss Crossbeam-340 SEM), with a built-in energy dispersive X-ray (EDX) spectrometer, was used. The operating voltage of the SEM was 1.20 kV, with a beam width of 3.6 mm and magnification of 5000 KX. Additionally, for the 3-dimensional surface features’ analyses, an atomic force microscope (AFM) model (Veeco Dimension-3100 AFM) was used. The scanning frequency of the AFM to scan the film surface was kept as low as around 0.90 Hz, and the mode was set to non-contact to avoid any damage to the surface of the samples. An X-ray diffraction (XRD) instrument (Smartlab, Rigaku X-ray diffractometer) was employed with a wavelength source of CuK α = 1.54° to determine the crystallinity and phase analysis of the PANI/ZnO. For the photovoltaic (PV) and current–voltage (I-V) measurements of the fabricated ITO/P3HT/PANI/ZnO/Ag devices, a Fytronix solar simulator connected with a source measure unit (SMU) was used under standard testing conditions (STC) at 1.5 AM filters, 25 °C, and 100 mW/cm^2^ irradiation and dark conditions, respectively.

## 3. Results

### 3.1. Material’s Characterization

**Figure 2:**
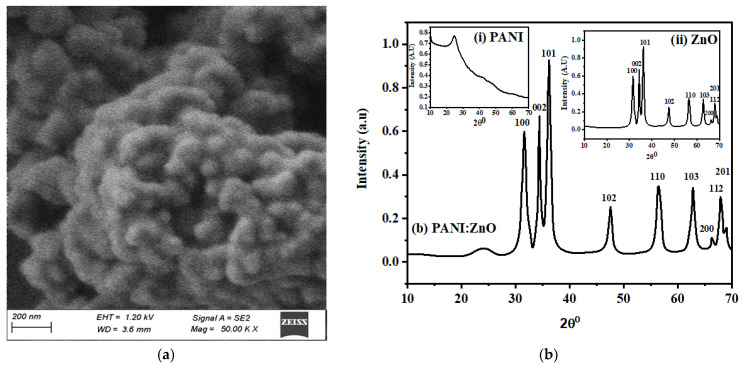

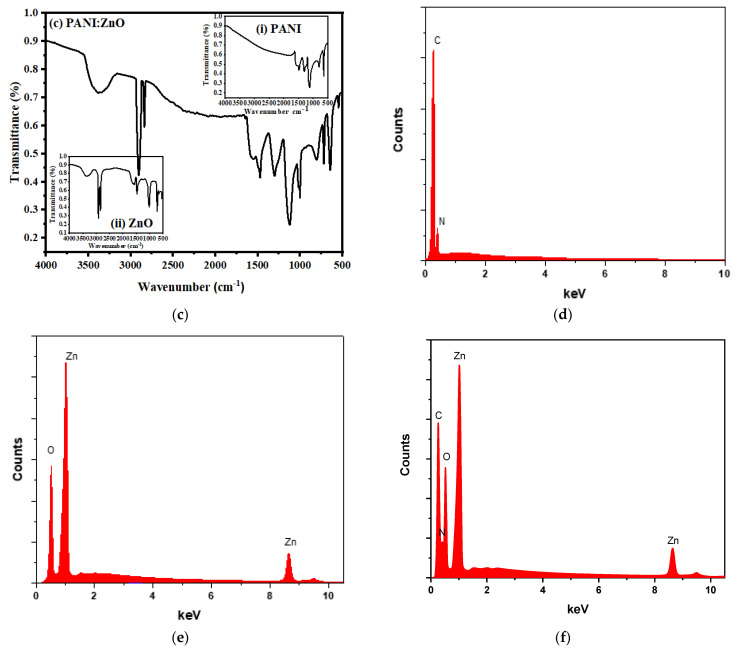
(**a**) SEM micrograph and (**b**) XRD spectrum of PANI/ZnO; inset, XRD of (i) pure PANI and (ii) pure ZnO. (**c**) FTIR transmission spectrum of PANI/ZnO; inset, FTIR of (i) pure PANI and (ii) pure ZnO. (**d**) EDS of PANI, (**e**) ZnO, and (**f**) PANI/ZnO nanocomposites.

The morphology of the PANI/ZnO thin film was analyzed by scanning electron microscopy, as shown in Figure 2a. These photos of the PANI nanocomposites indicate that the ZnO NPs presented in the matrix have a spherical form and prominent grain boundaries. As a result of PANI’s dominance, the nanocomposites have a clumped structure, so the agglomerated form is the result of the wurtzite ZnO nanoparticles. However, because PANI is amorphous, the blend was dominated by hexagonally wurtzite ZnO nanoparticles. In addition to demonstrating the existence of aggregated macromolecules, the SEM image of the composite shows that the ZnO nanoparticles are embedded in a polyaniline matrix [24]. Furthermore, there is some non-uniformity and grain aggregation in a few spots, which may be the result of uneven and rapid PANI/ZnO film deposition during the spin-coating technique. Due to the irregularity and roughness of the film, the charge carriers became localized as a result of charge-trapping and interfacial states.

The XRD technique was utilized to determine the type of prospective material, i.e., crystalline, polycrystalline, or amorphous. Figure 2b depicts the XRD spectrum of the PANI/ZnO thin film produced on the previously washed quartz glass at room temperature. The broad and weak peak at approximately 2θ = 24.63° is attributed to the PANI, suggesting a very low degree of crystallinity and considered amorphous due to its broad and wide peak, which consists of a long chain of alternative single and double bonds of carbon (C) and nitrogen (N), respectively. However, A. Nishara Begum et al. clarified the untreated and treated PANI and displayed the results accordingly. The results indicate that the crystallinity of PANI depends upon the heat treatment; however, the untreated samples show the PANI peak at 20.23°, with a crystallinity percentage of 36%. In the present work, the PANI peak at 2θ = 24.63° will further lessen the crystallinity percentage [25]. On a scale of 2θ, the angles of 31.62° 34.41°, 36.15°, 47.56°, 56.41°, 62.81°, 66.28°, 67.87°, and 68.98° are the most notable peaks, which belong to the (100), (002), (101), (110), (103), (200), (112), and (201) hexagonal ZnO crystal planes. All the peaks are characteristic of wurtzite (hexagonal) ZnO NPs (File No. 01-080-0075). Moreover, the average crystallite size, d-spacing, and dislocation density of the PANI/ZnO nanocomposites were measured to be 43.74 nm, 1.9562 nm, and 4.84 × 10^−4^, respectively. Because of the amorphous characteristics of PANI, it is clearly outmatched by the ZnO NPs in the XRD pattern of the PANI/ZnO nanocomposites [26].

The thin films of the PANI/ZnO nanocomposites were analyzed for their chemical structures and/or functional groups by using the FTIR spectrum. The PANI/ZnO thin films were exposed to IR radiation; additionally, the transitions between the vibrational energy levels were detected as a transmission spectrum at distinct wavenumbers (cm^−1^), as depicted in Figure 2c, and the details are listed in Table 1. The PANI/ZnO blend exhibits peaks at 3379 cm^−1^, 2908 cm^−1^, and 2840 cm^−1^, which correlate to the O-H stretching mode and C-H stretching, accordingly, because of the inclusion of the ZnO NPs. Likewise, the C=N and C=C stretchings of the quinoid ring are reflected in the 1646 cm^−1^ and 1546 cm^−1^ bands. The C-H bending spectrum (also known as the in-plane bending spectrum) peaks at 1475 cm^−1^, the PANI/ZnO peaks at 1305 cm^−1^, and the C-O stretching peaks at 1000 cm^−1^. The metal-oxide stretching vibrations and C-H out-of-plane bending in the benzenoid ring are revealed by the patterns at 810 cm^−1^, 721 cm^−1^, 647 cm^−1^, and 545 cm^−1^ [27].

Figure 2d–f depict the EDS spectra utilized to identify the elemental constitution of the PANI, ZnO, and PANI/ZnO nanocomposite thin films, respectively. The spectrum in Figure 2d exhibits the existence of pure PANI by indicating the presence of carbon and nitrogen only, without any extra elemental contamination. The spectrum further confirms the atomic and weight percent ratios of the C/N in the PANI film, which are 89.97:10.03 and 88.68:11.32, respectively. There are peaks at 0.15 keV and 0.20 keV, respectively, that correspond to carbon and nitrogen. Figure 2e shows the presence of Zn and O with no other elements, as exhibited by the peaks in the EDS. The atomic and weight percentage ratios of the Zn/O in the ZnO film are 51.05:48.95 and 81.36:18.64, respectively. All the peaks linked with the Zn and O are observed at 0.51 keV, 1.01 keV, 8.63 keV, and 9.50 keV, as shown in Table 2. From the EDS spectra, adding ZnO to the nanocomposites (NCs) increased the absorption intensity due to the uniform dosing of the ZnO in the NCs, as shown by the peak’s patterns.

**Figure 3:**
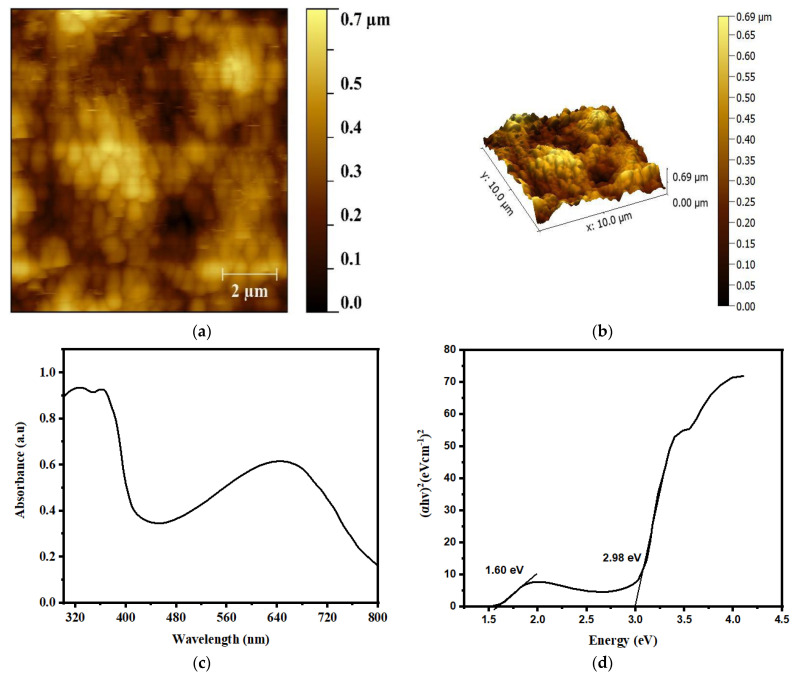
(**a**) 2D and (**b**) 3D AFM images of PANI/ZnO NCs; (**c**) UV-Vis absorption spectra and (**d**) energy bandgap of PANI/ZnO thin film.

The morphological characteristics of the PANI/ZnO nanocomposite (NC) thin films were analyzed by atomic force microscopy (AFM) to determine whether or not the addition of the ZnO NPs caused any structural alterations. Figure 3a,b depict 2D and 3D AFM images of the PANI/ZnO thin film, with a root mean square (RMS) and average roughness (*R_a_*) of 39.74 nm and 48.64 nm, using a scanning rate of 0.9 Hz and a scale size of 10 × 10 µm^2^ via the non-contact mode. In the 2D image, an agglomeration can be seen between the PANI and ZnO nanoparticles; however, the 3D image shows the presence of some mosaics and islands on the top of the PANI/ZnO thin film. According to the AFM analysis, the surface roughness was caused by the incorporation of ZnO into the polymeric matrix. Because of the availability of the folded ZnO sheets in the polymer matrix, the 3-dimensional inspection is rough. Therefore, it was revealed that the surface of the PANI/ZnO NCs is slightly roughened due to the incorporation of ZnO. The results indicate that the increased interaction between the nanoparticles and polymer is responsible for the observed surface morphological variations. A higher surface roughness presumably results in noticeably efficient devices, as rough and non-uniform films are preferable for obtaining more light because of the optimized photo absorption and reduced reflection from the film’s surface.

Figure 3b depicts the UV-Vis spectra from which the optical absorption width and bandgap of the PANI/ZnO blend were estimated. PANI’s electronic spectrum is divided into two distinct bands, referred to as the Soret or B-band and Q-band, because of its highly conjugated π-orbitals. The former band normally exists in the UV region, with the considerably higher energy having strong peaks, whilst the latter usually exists in the visible region, with relatively weak peaks. However, due to the π-π* transitions from the ground state (S_0_) to the second or higher excited states (S_2_ or S_n_) and the inclusion of the ZnO NPs in the blend, the absorption spectra of the PANI/ZnO film exhibit two modest but eminent peaks in the Soret or B-band, at 326 and 362 nm, respectively. The peak at 362 nm, on the other hand, indicates that the nanocomposite blend is equally dispersed with the ZnO nanoparticles. At 649 nm, the Q-band absorption peak of the PANI/ZnO demonstrates the π-polaron transition of the polyaniline (PANI)-conjugated ring structure. The findings depicted in Figure 3c are in good accordance with those published elsewhere [28].

By using the following Tauc’s relation [29], the optical bandgap of PANI/ZnO is calculated as:

α*E* = *A*(*E* − E_g_)^*θ*^ (1)

where E_g_ shows the energy bandgap, *E* is the incident photon energy, *θ* is a transition constant, and *α* is the absorption coefficient, which can be computed as:

(2) $$A=-\ln\left(\frac{I}{I_{0}}\right)$$

(3) A=αd

Consequently, *A* is the absorbance, whereas *d* indicates the film thickness of the fabricated devices. For an indirect transition between the energy levels, the transition constant is 2, whereas, for a direct transition, it is 1/2. The direct transition model is used to determine the value of E_g_ for PANI/ZnO devices due to the direct bandgap property of these semiconductors. Using the relationship between (*αE*^2^) and *E*, illustrated in Figure 3d, we can calculate E_g_ using Equation (1). This relationship is expressed as the intercept of the slope of the linear areas in the curve at the zero y-axis. Two E_g_ values of 1.60 and 2.96 eV were found for the PANI/ZnO, which is more consistent with what has been published in the literature [28].

### 3.2. Current–Voltage (I-V) Characteristics

#### 3.2.1. I-V Parameters in the Dark

**Figure 4:**
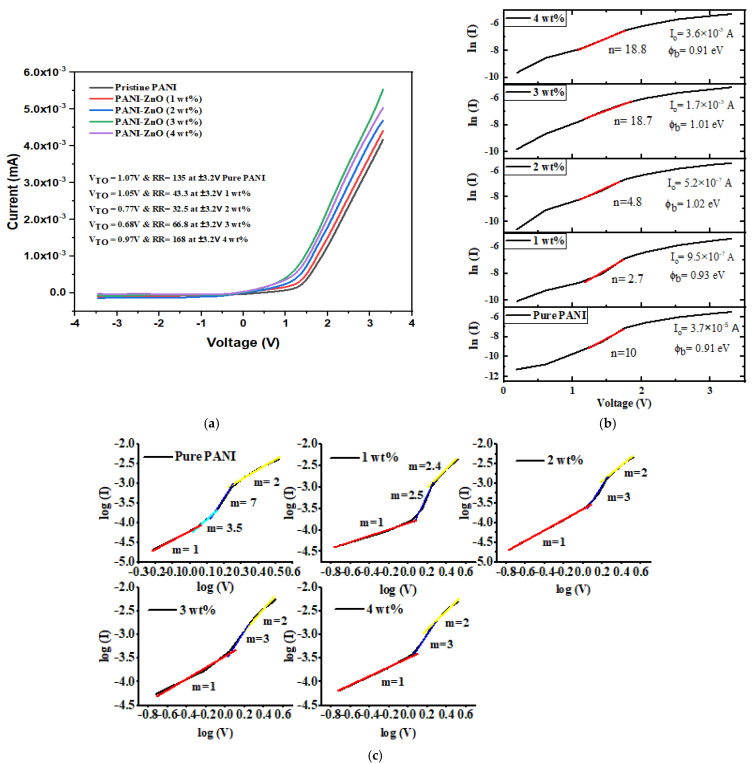
(**a**) I-V graph (in the dark), (**b**) semi-log I-V graph, and (**c**) log I-log V curve of pure PANI and four different concentrations of ZnO as an ITO/P3HT/PANI/ZnO/Ag heterojunction.

To identify and comprehend the heterojunction/interfacial properties, the I-V measurements of the ITO/P3HT/PANI/ZnO/Ag and its four distinct wt% devices in dark conditions at 25 °C were examined. The ITO/P3HT/PANI/ZnO/Ag devices are rectifying and non-ohmic due to the asymmetrical I-V curve depicted in Figure 4a. However, for the pure and different amounts of ZnO in the polymer matrix, the current rectification ratio (RR) and turn-on voltage (V_TO_) were measured, and the data are given in Table 3. Between the PANI/ZnO layer and the ITO, the interfacial states, series resistance (R_s_), and depletion region/barrier height (b) had a big effect on the exponential behavior of the I-V characteristics in the forward voltage region, even though the ideality factor (n), which gave enough information and understanding about how the device works, defined the quality of the interface/heterojunction. On the other hand, the quantity of the minority charge carriers across the heterojunction in the reverse bias was determined by the reverse saturation current. The estimated value of the R_s_ and shunt resistance (R_sh_) for pure PANI and four various concentrations of ZnO with PANI are shown in Table 3 and were obtained from the voltage (V) vs. the dV/dI (R_j_) relation, correspondingly. The performance of a solar cell is typically greater when the R_s_ is much smaller than the R_sh_, i.e., R_s_ ≪ R_sh_. The R_s_ is an unwanted and invasive characteristic that actually restricts the current through the heterojunction and lowers the value of the RR of the device in dark situations. Additionally, this parasitic influence of the R_s_ has a negative impact on the value of n, which decreases the effectiveness of ITO/P3HT/PANI/ZnO/Ag devices. The internal interface layers between the P3HT and PANI/ZnO, lateral conduction via transparent-conducting oxide ITO, and Ag contact to establish the R_s_ all play a role in the P3HT/PANI/ZnO/Ag solar cell. Additionally, the primary purpose of series resistance in solar cells is to lower the fill factor (FF), while very high values of R_s_ can also lower the short-circuit current (I_sc_). Because the R_s_ has all of these bad effects, it is called a parasitic parameter. Still, the values of R_s_ 1 kΩ and R_s_ 2 kΩ found for all of the concentrated devices were lower than those found for other organic–inorganic semiconductors that have been reported before [29,30]. Table 4 presents the comparison of the data obtained from the optimized PANI/ZnO blend with the previously published work. Using the log-linear scale (ln(I)-V) of the I-V characteristics presented in the inset of Figure 4b, we can determine the other essential junction parameters of the device, such as *n* and *I*_0_, with the help of Equations (4)–(7).

(4) $$I=I_{0}\left[exp\left(\frac{q\left(V-IR_{s}\right)}{nkT}\right)-1\right]$$

Here, $I_{0}$ is the reverse saturation current, *q* is the electronic charge, *V* is the applied voltage, *R_s_* is the series resistance, *n* is the ideality factor, *k* represents the Boltzmann constant, and *T* is the absolute temperature.

(5) $$I_{0}=AA^{*}T^{2}exp\left(\frac{-q\phi_{b}}{kT}\right)$$

where *A*, A*, and *ϕ_b_* are the active areas of the device, the Richardson constant (A* = 1.3 × 10^5^ Acm^−2^ K^−2^ for the ITO substrate), and the barrier height, respectively.

(6) $$n=\frac{q}{kT}\frac{dV}{d\left(\ln I\right)}$$

(7) $$\phi_{b}=\frac{kT}{q}\ln\left(\frac{AA^{*}T^{2}}{I_{o}}\right)$$

The charge conduction mechanism in the ITO/P3HT/PANI/ZnO/Ag heterojunction can be described by using the Power law/Child’s law, i.e., as expressed in Figure 4c. Figure 4b shows a linear part of the curve with a slope of m, and the different values of m point to different conduction mechanisms. When m = 1, an ohmic conduction mechanism is assumed because the current cannot be rectified by applying voltage. The heterojunction’s asymmetric I-V characteristics and current rectification are caused by the space charge-limiting current (SCLC) region, which is indicated by the change in the relation at m = 2. When m is more than 2, it reveals a zone of a trapped charge-limiting current (TCLC). Trapped charge-limiting current (TCLC) regions are created at m > 2, and these regions are the result of many shallow and/or deep traps at the heterojunction that limit the amount of current that may flow through the device. For the ITO/P3HT/PANI/ZnO/Ag and the four different wt% devices, the logI-logV graph in Figure 4c shows almost three different regions, i.e., I, II, and III, which correspond to the ohmic conduction process at m = 1, the SCLC conduction mechanism at m = 2, 2.4, or 2.5, and the TCLC process at m = 3, 3.5, or 7. Figure 4a depicts the rectifying behavior of the ITO/P3HT/PANI/ZnO/Ag and its various varying concentrated devices. Consequently, the SCLC is the dominating conduction mechanism [19]. In the SCLC, the density of the inserted charge carriers is substantially larger than the density of the free charge carriers produced thermally, which causes the current’s rectification.

**Table 3:** Dark I-V parameters of prepared devices.

Prepared Devices	ZnO (wt%)	V_TO_ (V)	RR at (V)	n	I_0_ (mA)	ϕ_b_ (eV)	R_sh_	R_s_
Pure PANI	0	1.07	135 at ±3.2	10	3.7 × 10^−5^	0.91	0.13 GΩ	2 kΩ
PANI/ZnO	1	1.05	43.3 at ± 3.2	2.7	9.5 × 10^−7^	0.93	65 kΩ	2 kΩ
PANI/ZnO	2	0.77	32.5 at ± 3.2	4.8	3.7 × 10^−7^	1.02	69 kΩ	2 kΩ
PANI/ZnO	3	0.68	66.8 at ± 3.2	18.7	1.7 × 10^−5^	1.01	76 kΩ	1 kΩ
PANI/ZnO	4	0.97	168 at ± 3.2	18.8	3.6 × 10^−5^	0.91	0.13 GΩ	2 kΩ

**Table 4:** Comparison of Dark I-V Characteristics with reported work.

Prepared Devices	n	I_0_ (mA)	R_s_	References
VONc/ZnO	1.18	5.98 × 10^−10^	16.3 kΩ	[31]
PANI/n-Si	1.32	5 × 10^−4^	3.11 MΩ	[32]
NiPc/p-Si	1.51	1.2 × 10^−8^	750 kΩ	[33]
PANI/ZnO	18.7	1.7 × 10^−5^	1 kΩ	Present

#### 3.2.2. Illumination of I-V Characteristics

The assessment of an OSC’s performance and the optimization of its design and fabrication require an in-depth understanding of the electrical properties from the current–voltage characteristics. Numerous losses related to parameters, such as the Isc, FF, and Voc, can be determined in the PCE of the OSCs. Furthermore, the n, R_s_, R_sh_, and I_0_, the basic device parameters, are the causes that can regulate the losses. The R_sh_ occurs in solar cells due to manufacturing defects and provides an alternate path for the charge carriers produced by photons to travel.

**Figure 5:**
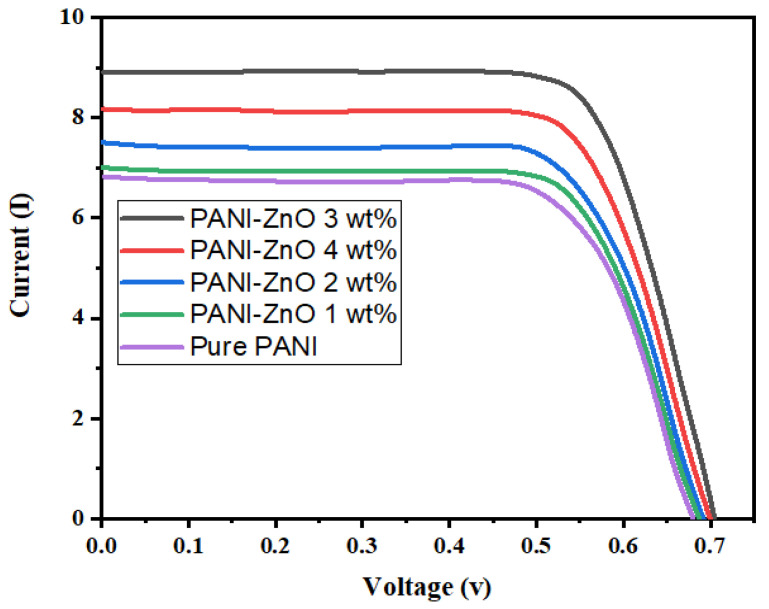
I-V graph (under Illumination) for Pure PANI and four different concentrations of ZnO as ITO/P3HT/PANI/ZnO/Ag heterojunction.

ITO/P3HT/PANI/ZnO/Ag and its four different wt% devices’ photovoltaic measurements are displayed as a current density (J) vs. a voltage graph, as depicted in Figure 5, where the STC is specified as 100 mW/cm^2^, AM 1.5 G, and 25 °C. The graph demonstrates that the photocurrent flowing across the device is greater in the light than in the dark. The greater value of J is responsible for the generation of excitons (electron-hole pairs) in the absorber layer (the PANI/ZnO film) that detach into individual electrons and holes at the heterojunction interface, resulting in an abundance of photocurrent. However, the quantitative values of the fill factor (FF) and PCE are derived by the following relations to assess the photovoltaic performance of the ITO/P3HT/PANI/ZnO/Ag and four different wt.% of ZnO devices.

In Equation (8), V_max_, J_max_, V_oc_, and J_sc_ represent the maximum voltage, maximum current density, open circuit voltage, and short circuit current, correspondingly; however, E indicates the solar irradiance, and A is the active area of the device. Additionally, the respective values of V_oc_, J_sc_, FF, and PCE are determined and listed in Table 5. Although, the higher PCE from Equation (9) for the ITO/P3HT/PANI/ZnO (3 wt%)/Ag device is determined to be 4.48 ± 0.5%.

**Figure 6:**
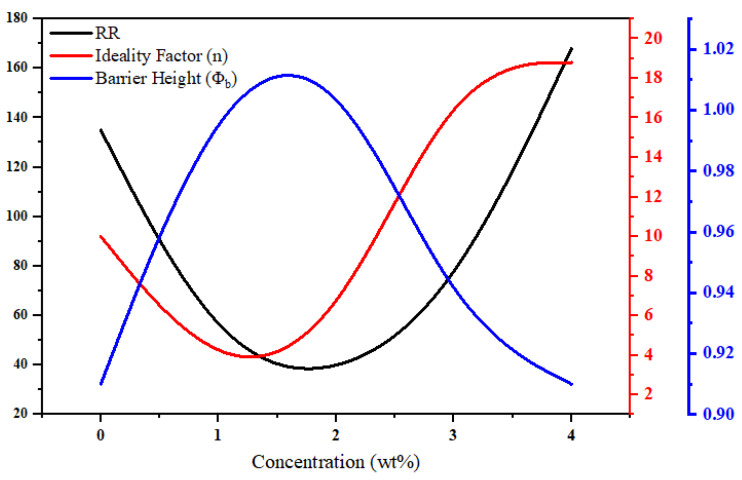
A depiction of the effects of the ZnO concentration (wt%) against the RR, ideality factor, and barrier height for the devices.

Herein, we also report the effect of different concentrations (wt%) of ZnO on the rectification ratio, ideality factor, and barrier height shown in Figure 6. According to Equation (8), it can be seen that $\varphi_{b}$ and n are inversely proportional to each other, which means that with an increase in the value of “n”, the value of $\phi_{b}$ is higher for our fabricated ITO/P3HT/PANI/ZnO/Ag device. In a similar way, from Equation (8), it can be analyzed that $\phi_{b}$ increases so that the leakage of I_0_/I_s_ exponentially increases. Consequently, as we can see in Equation (9), when I_0_ decreases, which is due to the minority carriers, the RR increases. The reason for this increase may be attributed to the less recombination of the charge carriers passing through the depletion region. A similar behavior is obtained for the different fabricated devices.

(8) $$I_{D}=AA^{*}T^{2}e^{\frac{-\varphi_{b}}{nkT}}$$

(9) $$\mathrm{RR}\sim \frac{1}{I_{0}}\sim \frac{1}{exp\left(\frac{-\varphi_{b}}{nkT}\right)}$$

Now, when the concentration of ZnO increases, initially, the recombination of PANI and ZnO takes place at a higher rate; however, with a further increase in concentration, this recombination process accelerates due to the more ZnO-contributed charge carriers, which, as a result, increase the RR and n, adversely decreasing $\phi_{b}$.

In comparison with the previously published organic–inorganic heterostructure solar cells, PANI/ZnO-based devices showed a remarkable PCE of 4.48%, as compared to the other structures mentioned in Table 6.

## 4. Conclusions

In this work, an organic–inorganic semiconductor (PANI/ZnO) nanocomposite—which combinedly developed a donor/acceptor blend—was used as an active photo-absorber layer for the fabrication of hybrid solar cells. In bulk heterojunction solar cells, one of the critical parameters was the interfacial area between the donor and acceptor materials, which can be tuned by varying the concentration (wt.%) of ZnO in the PANI as to obtain the maximum possible junctions’ area available to the excitons. For this reason, the various wt.% of ZnO in the PANI resulted in different photovoltaic parameters and, consequently, the power conversion efficiency (PCE). However, the optimum PANI/ZnO ratio was found to be 1:3 wt.%, which is attributed to the provision of the large area of the junctions throughout the volume of the PANI/ZnO blend, which further led to better charge separation and less recombination. Moreover, when the concentration of the ZnO nanoparticles was further increased to 4 wt.%, the PCE dropped drastically, which was attributed to the agglomeration of the ZnO nanoparticles. Due to the agglomeration, the ZnO gradually lost its large surface-to-volume ratio that, consequently, decreased the interfacial area throughout the bulk layer between the PANI and ZnO. The value of the PCE at the optimized concentration (3 wt.%) was measured to be 4.48 ± 0.5%, which was better than that measured for the pristine PANI (3.10 ± 0.5%). The current–voltage properties of the ITO/P3HT/PANI/ZnO/Ag device were measured in dark conditions as well to quantify the quality of the heterojunction formed between the PANI and ZnO. In dark conditions, the series resistance was measured as ~1 kΩ, which was sufficiently low enough to ensure the high PCE of the solar cells. The present study suggests the potential of organic–inorganic hybrid nanocomposites at their optimized conditions for high performance photovoltaic applications.

## Figures and Tables

**Table 1:** Vibrational bond dynamics in PANI/ZnO thin film.

Peaks (cm^−1^)	Bonds Nature/Dynamics
545	Zn-O Stretch Vibration
647	C-H Out-of-Plane Bend in Benzenoid Ring
721	C-H Out-of-Plane Bend in Benzenoid Ring
810	C-H Out-of-Plane Bend in Benzenoid Ring
1000	C-O Stretch
1305	PANI/ZnO Spectra
1475	C-H Bend or In-Plane Bend
1546	C=C Stretch of Quinoid Ring
1646	C=N Stretch of Quinoid Ring
2840	C-H Stretch
2908	C-H Stretch
3375	O-H Stretch

**Table 2:** EDX elemental analysis of PANI, ZnO, and PANI/ZnO nanocomposite thin films.

Material	Element	Weight %	Atomic %
PANI	C K	88.86	89.97
	N K	11.32	10.03
ZnO	O K	18.64	48.95
	Zn L	81.36	51.05
PANI/ZnO	C K	40.12	44.86
	N K	7.48	6.62
	O K	18.31	48.08
	Zn L	33.56	21.05

**Table 5:** Light I-V characteristics of the prepared devices.

Prepared Devices	I_SC_ (mA)	V_OC_ (V)	FF (%)	P_Max_ (W)	η (%)
Pure PANI	6.8	0.68	67	3.14	3.10 ± 0.5
PANI:ZnO 1 wt%	7	0.68	65	3.13	3.09 ± 0.5
PANI:ZnO 2 wt%	7.5	0.69	69	3.57	3.57 ± 0.5
PANI:ZnO 3 wt%	8.9	0.7	72	4.48	4.48 ± 0.5
PANI:ZnO 4 wt%	8.21	0.7	71	4.09	4.08 ± 0.5

**Table 6:** Comparison of present work with that previously reported elsewhere.

Device Structure	Matrix	Dopant	PCE (%)	References
ITO/PEDOT:SS/P3HT:ZnO(1:1)/Al	P3HT	ZnO	2.0	[34]
ITO/PEDOT:SS/PCPDTBT:CdSe(9:1)/Lif/Al	PCPDTBT	CdSe	3.20	[35]
ITO/PEDOT:PSS/P3HT:PC_71_BM:ZnO NS(1:0.8:3)/LiF/Al	P3HT:PC_71_BM	ZnO	3.81	[36]
ITO/P3HT/PANI/ZnO(1:3)/Ag	PANI	ZnO	4.48	Present work

## Data Availability

Not applicable.
